# Outbreak of Serotype 1 Invasive Pneumococcal Disease, Kibera Urban Informal Settlement, Nairobi, Kenya, 2023

**DOI:** 10.3201/eid3102.241652

**Published:** 2025-02

**Authors:** Terry Komo, Patrick K. Munywoki, Maria da Gloria Carvalho, Joshua Auko, Alice Ouma, Allan Audi, George O. Agogo, Daniel Omondi, Arthur Odoyo, Herine Odiembo, Newton Wamola, Mike Osita, Clayton Onyango, Naomi Lucchi, Peninah Munyua, Amy Herman-Roloff, Shanda Larson, Sopio Chochua, Fabiana C. Pimenta, Godfrey Bigogo, Jennifer R. Verani

**Affiliations:** Kenya Medical Research Institute, Kisumu, Kenya (T. Komo, J. Auko, A. Ouma, A. Audi, D. Omondi, A. Odoyo, H. Odiembo, N. Wamola, M. Osita, G. Bigogo); US Centers for Disease Control and Prevention, Nairobi (P.K. Munywoki, G.O. Agogo, C. Onyango, N. Lucchi, P. Munyua, A. Herman-Roloff); US Centers for Disease Control and Prevention, Atlanta, Georgia, USA (M.D.G. Carvalho, S. Larson, S. Chochua, F.C. Pimenta, J.R. Verani)

**Keywords:** Streptococci, Streptococcus pneumoniae, bacteria, respiratory infections, pneumococcal diseases, outbreak, 10-valent pneumococcal vaccine, vaccines, Kibera, Nairobi, Kenya

## Abstract

Use of 10-valent pneumococcal conjugate vaccine in Kenya has led to substantial reductions in vaccine-type pneumococcal carriage and invasive pneumococcal disease. However, analysis of recent surveillance data indicates an outbreak of vaccine-type serotype 1 in 2023 in Kibera, Kenya. Continued monitoring of invasive pneumococcal disease in Kenya is warranted.

*Streptococcus pneumoniae* (pneumococcus) is a leading cause of pneumonia, sepsis, and meningitis and is most prevalent in resource-poor settings (*1*). Pneumococcal serotype 1 (ST1) is an important cause of disease, particularly in sub-Saharan Africa; it is highly invasive and infrequently detected in carriage (*2*). Pneumococcal conjugate vaccines (PCVs) protect against vaccine-type disease and carriage among vaccinated persons (direct effects), leading to decreased transmission of vaccine serotypes and reduced disease among unvaccinated persons (indirect effects) (*3*). All currently available PCVs protect against ST1. 

In 2011, Kenya introduced 10-valent PCV (Synflorix; GlaxoSmithKline, https://www.gsk.com) (PCV10-GSK), which is administered in 3 doses, at 6, 10, and 14 weeks of age. Vaccine-type invasive pneumococcal disease (IPD) subsequently declined by ≈70%–90%, and vaccine-type carriage declined by ≈50%–60% (*3*). In 2022, Kenya switched to a newer 10-valent PCV (Pneumosil; Serum Institute of India, https://www.seruminstitute.com) (PCV10-SII).

## The Study

The Population-Based Infectious Disease Surveillance (PBIDS) platform is implemented in defined catchment populations in Kibera, an urban informal settlement in Nairobi County, and Asembo, a rural area in Siaya County (*4*); the Kibera site includes ≈25,000 persons in an area ≈1 km^2^. Through household visits, we collected data on demographics, healthcare use, and vaccination. In both sites, PCV coverage among children <5 years of age has been stably >90%. At centrally located surveillance health facilities (an outpatient clinic in Kibera and an outpatient clinic with small inpatient ward in Asembo), PBIDS participants of all ages meeting standardized criteria for acute febrile illness or severe acute respiratory illnesses (SARI) undergo blood culture; SARI case-patients additionally have a nasopharyngeal swab collected for culture to monitor pneumococcal carriage. The PBIDS protocol was approved by the Kenya Medical Research Institute and the US Centers for Disease Control and Prevention. We obtained written informed consent from participants before sample collection.

We used standard culture procedures to identify pneumococcus. We performed serotyping by real-time PCR, Quellung reaction, or both. At the US Centers for Disease Control and Prevention, we conducted antibiotic susceptibility testing (AST) through broth microdilution (*5*) for ST1 IPD and carriage isolates from 2023 and short-read whole-genome Illumina sequencing (*6*). We identified single-nucleotide polymorphisms (SNPs) for core genomes by using kSNP3.0 with k-Mer size of 19 (*7*) and generated pairwise comparisons by using MEGA version 7 (*6*,*8*).

We calculated crude incidence rates and 95% CIs by dividing the number of ST1 bacteremia cases by person-years of observation (PYO). We adjusted incidence rates to account for missed blood samples and healthcare seeking (i.e., cases of medically attended acute febrile illness or SARI reported during household visits for which care was sought at a nonsurveillance facility). We calculated 95% CIs for adjusted incidence rate estimates by using Monte Carlo simulations (0.025 and 0.975 quantiles of 10,000 simulations), sampling from Poisson distribution for crude incidence, and binomial distribution for adjustment factors (*9*).

We expressed overall and serotype-specific pneumococcal carriage among SARI cases as a percentage, excluding those with limited growth of any organisms, which we deemed poor-quality specimens. We examined ST1 IPD and carriage occurring during January 1, 2018–August 20, 2024. For ST1 isolates from 2023 in Kibera, we described sequence types and AST.

**Figure 1:**
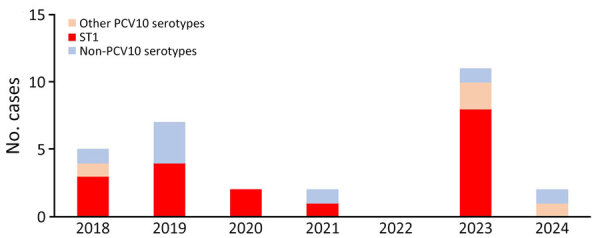
Serotype distribution among invasive pneumococcal disease cases, Kibera Urban Informal Settlement, Nairobi, Kenya, January 1, 2018–August 20, 2024. Other PCV10 serotypes are those common to Synflorix 10-valent PCV (GlaxoSmithKline, https://www.gsk.com) and Pneumosil 10-valent PCV (Serum Institute of India, https://www.seruminstitute.com) (5, 6B, 7F, 9V, 15, 19F, and 23F), Synflorix unique (4 and 18C), and Pneumosil unique (6A and 19A). PCV10, 10-valent pneumococcal conjugate vaccine; ST1, serotype 1.

Among 4,913 blood samples collected in Kibera, 149 (3.0%) had bacteria isolated, of which 30 (20.1%) were *S. pneumoniae*. ST1 accounted for 18/30 (60.1%) pneumococcal isolates; 8/18 (44.4%) ST1 isolates were collected in 2023 (Figure 1; Appendix). The crude ST1 IPD incidence rate during 2018–2022 was 8.7 cases/100,000 PYO (95% CI 4.7–16.2 cases/100,000 PYO) versus 35.5 cases/100,000 PYO (95% CI 17.7–71.0 cases/100,000 PYO) in 2023 (Appendix). The adjusted ST1 IPD incidence rate during 2018–2022 was 17.9 cases/100,000 PYO (95% CI 7.3–30.1 cases/100,000 PYO) versus 66.8 cases/100,000 PYO (95% CI 24.8–118.7 cases/100,000 PYO) in 2023 (Appendix). All ST1 IPD case-patients in 2023 were children 2–10 years of age (median 4.5 years of age), and all of them were age-eligible for PCV10-GSK; 5/8 (62.5%) were fully immunized (Table 1). No hospitalizations or fatalities occurred among ST1 case-patients.

**Table 1:** Characteristics of IPD cases, Kibera Urban Informal Settlement, Nairobi, Kenya, January 1, 2018–August 20, 2024*

Characteristic	Serotype and surveillance period		
	ST1, 2023	ST1, 2018–2022	Non-ST1, 2018–2024
No. cases	8	10	12
Median age of patient, y (range)	4.5 (2.5–9.7)	12 (0.79–36.6)	10.6 (0.44–45.5)
Sex of patient			
M	6 (75.0)	5 (50.0)	8 (66.7)
F	2 (25.0)	5 (50.0)	4 (33.3)
Age eligibility for PCV			
Age-eligible for PCV10-GSK	8 (100)	5 (50.0)	7 (58.3)
Age-eligible for PCV10-SII	0	0	2 (16.7)
Not age-eligible for PCV	0	5 (50.0)	3 (25.0)
Vaccination status known	8 (100)	2 (40.0)	7 (77.8)
0 PCV doses	1 (12.5)	0	3 (42.9)
1 PCV dose	1 (12.5)	1 (50.0)	0
2 PCV doses	1 (12.5)	0	1 (14.2)
3 PCV doses	5 (62.5)	1 (50.0)	3 (42.9)
Month of detection			
January	0	1 (10.0)	0
February	0	3 (30.0)	2 (16.7)
March	2 (25.0)	2 (20.0)	0
April	3 (37.5)	1 (10.0)	2 (16.7)
May	0	0	2 (16.7)
June	1 (12.5)	1 (10.0)	1 (8.3)
July	0	0	0
August	0	0	2 (16.7)
September	0	1 (10.0)	0
October	1 (12.5)	1 (10.0)	0
November	1 (12.5)	0	1 (8.3)
December	0	0	2 (16.7)
Disposition			
Discharged home from clinic	8 (100)	10 (100)	12 (100)
Alive at 1 month after IPD†	8 (100)	10 (100)	12 (100)

*Values are no. (%) except as indicated. IPD, invasive pneumococcal disease; PCV, pneumococcal conjugate vaccine; PCV10-GSK, Synflorix 10-valent PCV (GlaxoSmithKline, https://www.gsk.com); PCV10-SII, Pneumosil 10-valent PCV (Serum Institute of India, https://www.seruminstitute.com); ST1, serotype 1. †Vital status at 1 month after date of blood culture was verified from household demographic data.

**Figure 2:**
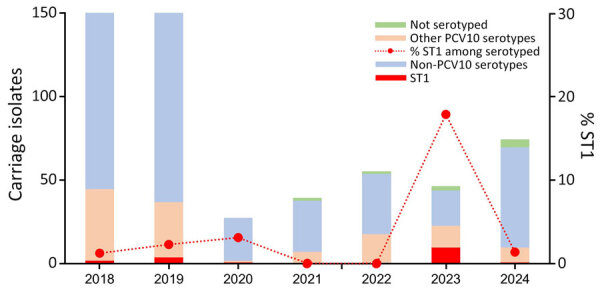
Serotype distribution among invasive pneumococcal disease carriage isolates, Kibera Urban Informal Settlement, Nairobi, Kenya, January 1, 2018–August 20, 2024. Other PCV10 serotypes are those common to Synflorix 10-valent PCV (GlaxoSmithKline, https://www.gsk.com) and Pneumosil 10-valent PCV (Serum Institute of India, https://www.seruminstitute.com) (5, 6B, 7F, 9V, 15, 19F, and 23F), Synflorix unique (4 and 18C), and Pneumosil unique (6A and 19A). PCV10, 10-valent pneumococcal conjugate vaccine; ST1, serotype 1.

Among serotyped carriage isolates from SARI cases during 2018–2022, 1.5% (7/464) were ST1, and frequency by year ranged from 0 to 3.1% (Table 2; Figure 2). In 2023, 17.9% (10/56) of serotyped carriage isolates from SARI cases in Kibera were ST1.

**Table 2:** Enrollment of SARI cases, pneumococcal carriage testing, and serotyping, Kibera Urban Informal Settlement, Nairobi, Kenya, January 1, 2018–August 20, 2024*

Year	SARI cases	NPs collected and tested	Poor growth†	Pneumococcus isolated‡	Serotyped	ST1	Other PCV10 types§	Non-PCV10 types	Serotype pending
2018	264	233 (88.3)	4 (1.7)	161/229 (70.3)	161 (100)	2 (1.2)	43 (26.7)	115 (71.4)	0
2019	318	312 (98.1)	0	174/312 (55.8)	174 (100)	4 (2.3)	33 (19.0)	137 (78.7)	0
2020	78	73 (93.6)	1 (1.4)	32/72 (44.4)	32 (100)	1 (3.1)	1 (3.1)	25 (78.1)	0
2021	133	110 (82.7)	11 (10.0)	40/99 (40.4)	39 (97.5)	0	7 (18.0)	31 (79.5)	1 (2.5)
2022	113	106 (93.8)	4 (3.8)	59/102 (57.8)	58 (98.3)	0	18 (31.0)	36 (62.1)	1 (1.7)
2023	125	120 (96.0)	11 (9.2)	58/109 (53.2)	56 (96.6)	10 (17.9)	13 (23.2)	21 (37.5)	2 (3.4)
2024	222	190 (85.6)	14 (7.4)	78/176 (44.3)	74 (94.9)	1 (1.4)	9 (12.2)	60 (81.1)	4 (5.1)

*Values are no. (row %) except as indicated. NP, nasopharyngeal swab sample; PCV10, 10-valent pneumococcal conjugate vaccine; PCV10-GSK, Synflorix 10-valent PCV (GlaxoSmithKline, https://www.gsk.com); PCV10-SII, (Pneumosil 10-valent PCV (Serum Institute of India, https://www.seruminstitute.com); SARI, severe acute respiratory illness; ST1, serotype 1. †Poor growth defined as <10 colonies of any bacteria. ‡Excludes samples with poor growth. §Includes serotypes common to PCV10-GSK and PCV10-SII (5, 6B, 7F, 9V, 15, 19F, and 23F), PCV10-GSK unique (4 and18C), and PCV10-SII unique (6A and 19A).

As of August 20, 2024, we had detected no ST1 IPD cases in 2024, and only 1.3% (1/74) of serotyped carriage isolates were ST1 (Tables 1, 2; Figures 1, 2). We observed no clear increase of ST1 in carriage or IPD in Asembo during the study period (data not shown).

We performed sequencing and AST for 13 ST1 isolates from 2023 (5 IPD and 8 carriage); 9 (69.2%) were sequence type 217, and 4 (30.8%) were sequence type 6056. Genomic analysis demonstrated a 4–15 SNP difference among sequence type 217 isolates and a 6–8 SNP difference among sequence type 6056 isolates, indicating close genomic relatedness. All isolates tested were susceptible to amoxicillin, cefuroxime, and erythromycin; had intermediate susceptibility to penicillin (nonmeningitis oral breakpoint); and were resistant to cotrimoxazole.

## Conclusions

Although PCV10-GSK in the Kenya routine infant immunization program has led to substantial reductions in vaccine-type pneumococcal carriage and disease, the surveillance data indicate an outbreak of vaccine-type ST1 in 2023 in Kibera. The IPD case counts from the surveillance area are small, which limits statistical power. However, the ≈4-fold increase in crude and adjusted ST1 IPD incidence rates in 2023 compared with the preceding 5 years, the >10-fold increase in ST1 carriage prevalence (reflecting increased transmission in the community), and the close genetic relatedness of sequenced ST1 isolates from 2023 are consistent with an outbreak. Furthermore, the predominant sequence type detected has been associated with pneumococcal meningitis outbreaks in Malawi (*2*) and Ghana (*10*). Pneumococcal disease outbreaks often occur in crowded, closed environments (*11*); Kibera is densely populated, and the data highlight the risk for IPD outbreaks in urban informal settlements.

ST1 is an important cause of pneumococcal disease outbreaks in sub-Saharan Africa (*2*). Although PCV implementation has reduced ST1 disease globally (*12*), several countries, particularly in the meningitis belt, have experienced ST1 IPD outbreaks even in the context of mature PCV programs with high coverage (*2*). The World Health Organization recommends 3-dose PCV schedules: either 3 primary doses and no booster or 2 primary doses and 1 booster. Booster doses can extend the duration of protection among vaccinated persons. The longer period of direct protection against vaccine serotypes might result in less circulation and more robust indirect protection, particularly against ST1, which frequently affects age groups beyond infancy (*13*,*14*). Kenya uses 3 primary doses and no booster schedule; however, if ST1 continues to pose a threat despite high PCV coverage, consideration of switching to 2 primary doses and 1 booster might be merited (*14*,*15*). Our data also highlight the importance of optimizing PCV coverage, given that ≈40% of ST1 case-patients in 2023 had not completed PCV vaccination.

Of note, although this outbreak was detected after Kenya switched PCV product from PCV10-GSK to PCV10-SII, all children with ST1 IPD in 2023 were born before 2022 and therefore eligible for PCV10-GSK. Thus, despite the temporal association, the observed increase in ST1 cases in Kibera in 2023 should not be considered a reflection of PCV10-SII performance in Kenya.

All ST1 IPD patients were managed as outpatients, and none died. All ST1 isolates with available AST data were susceptible to first-line treatment for nonsevere pneumonia in Kenya (https://www.researchgate.net/figure/Kenyan-Ministry-of-Health-MoH-guidelines-for-management-of-children-aged-2-59-months_tbl1_264744317).

Preliminary data from 2024 suggest that the ST1 outbreak in Kenya might have resolved. However, continued monitoring of IPD in Kibera and other parts of the country is warranted.

Appendix Additional information about outbreak of serotype 1 invasive pneumococcal disease, Kibera urban informal settlement, Nairobi, Kenya, 2023.
