# Supplementary material for: Outbreak of Serotype 1 Invasive Pneumococcal Disease, Kibera Urban Informal Settlement, Nairobi, Kenya, 2023
Source: Emerg Infect Dis. 2025 Feb;31(2):345–9. doi: 10.3201/eid3102.241652 (PMC11845131; doi:10.3201/eid3102.241652)
Supplement: Appendix — Additional information about outbreak of serotype 1 invasive pneumococcal disease, Kibera urban informal settlement, Nairobi, Kenya, 2023. [file 24-1652-Techapp-s1.pdf]

Article DOI: https://doi.org/10.3201/eid3102.241652

EID cannot ensure accessibility for supplementary materials supplied by authors. Readers who have difficulty accessing supplementary content should contact the authors for assistance.

Outbreak of Serotype 1 Invasive Pneumococcal Disease, Kibera Urban Informal Settlement, Nairobi, Kenya, 2023

Appendix

Appendix Table. Blood collection, pneumococcal testing, and incidence of serotype 1 (ST1) invasive pneumococcal disease in Kibera, January 1,2018–August 20, 2024

Year	Blood culture collected	SPN	ST1 cases	(PYO)	Crude incidence per 100,000 PYO	95% CI	ST1 cases*	ST1 cases†	Adjusted incidence per 100,000 PYO	95% CI
2018	759	5	3	22,689	13.2	4.3–41.0	3.8	7.3	32.0	0.0–73.4
2019	1,152	7	4	23,312	17.2	6.4–45.7	4.8	7.9	33.7	8.0–71.6
2020	452	2	2	23,465	8.5	2.1–34.1	2.3	3.7	15.6	0.0–41.5
2021	747	2	1	22,885	4.4	0.6–31.0	1.3	2.1	9.4	0.0–29.7
2022	501	0	0	22,715	0.0	–	0.0	0.0	0.0	–
2018–2022	3,611	16	10	115,066	8.7	4.7–16.2	12.4	20.6	17.9	7.3–30.1
2023‡	603	12	8	22,547	35.5	17.7–71.0	9.8	15.1	66.8	24.8–118.7
2024	699	2	0							

SPN, *Streptococcus pneumoniae;* ST1, Serotype 1; PYO, Person- years- observation; CI, Confidence interval.
*Cases adjusted for missed sampling.
†Cases adjusted for missed sampling and healthcare seeking
‡2023 row indicates the year with outbreak and contrast period.
